# Analysis of the functioning of temporary dialysis catheters in patients with COVID-19

**DOI:** 10.1590/1677-5449.202500052

**Published:** 2025-07-28

**Authors:** Bruno Jeronimo Ponte, Viviane Galli Dib, Arthur Souza Magnani, Felipe Soares Oliveira Portela, Marcela Juliano Silva Cunha, Lucas Lembrança Pinheiro, Nelson Wolosker

**Affiliations:** 1 Hospital Israelita Albert Einstein – HIAE, São Paulo, SP, Brasil.; 2 Faculdade Israelita de Ciências da Saúde – FICSAE, São Paulo, SP, Brasil.; 3 Universidade de São Paulo – USP, Faculdade de Medicina, São Paulo, SP, Brasil.

**Keywords:** COVID-19, central venous catheter, hemodialysis, catheter obstruction, COVID-19, cateter venoso central, hemodiálise, obstrução do cateter

## Abstract

**Background:**

Patients with acute renal failure requiring hemodialysis should be treated using temporary hemodialysis catheters due to the urgency and potential reversibility of the condition. To date, 3 studies in North America have suggested a higher risk of catheter-related issues in COVID-19 patients needing hemodialysis.

**Objectives:**

This study examines the functionality and complications of temporary hemodialysis access in COVID-19 patients at a Brazilian hospital during the coronavirus outbreak of 2020.

**Methods:**

A retrospective analysis was conducted at a COVID-19 referral center from May to July 2020. During this period, the Vascular Surgery team implanted temporary hemodialysis catheters in 107 patients. These patients were monitored, and their demographic and clinical characteristics were analyzed to identify any that correlated with catheter malfunction.

**Results:**

Of the 107 patients studied, 22 (20.6%) had catheter-related complications. Eighteen (16.8%) had catheter malfunctions, while 4 (3.7%) developed infections. Five patients had unfavorable clinical status and did not undergo catheter replacement. Catheter tip thrombosis was the cause of the malfunction in 13 of the patients with malfunctions. The analysis revealed that the only variables correlated with higher risk of malfunction were the need for orotracheal intubation (p = 0.009), deep vein thrombosis (p = 0.01), and a history of a previous catheter use (p = 0.002).

**Conclusions:**

The rate of temporary dysfunction of the high-flow catheter in patients with COVID-19 in this sample was similar to the rate described in the literature for patients without this disease.

## Resumo

**Contexto:**

Em geral, pacientes com insuficiência renal aguda que necessitam de hemodiálise utilizam cateteres temporários devido à urgência e à possível reversibilidade da condição. Até o momento, três estudos realizados na América do Norte indicam um risco maior de problemas relacionados ao cateter em pacientes com COVID-19 que precisam de hemodiálise.

**Objetivos:**

O presente estudo examina a funcionalidade e as complicações do acesso temporário para hemodiálise em pacientes com COVID-19 em um hospital brasileiro.

**Métodos:**

Foi realizada uma análise retrospectiva em um centro de referência para COVID-19 entre maio e julho de 2020. Durante esse período, a equipe de Cirurgia Vascular implantou cateteres temporários para hemodiálise em 107 pacientes. Os pacientes foram acompanhados, e as características demográficas e clínicas que poderiam estar correlacionadas ao mau funcionamento do cateter foram analisadas.

**Resultados:**

Dos 107 pacientes estudados, 22 (20,6%) apresentaram complicações relacionadas ao cateter. Dezoito (16,8%) apresentaram mau funcionamento, e quatro (3,7%) tiveram infecções. Cinco pacientes evoluíram com condições clínicas desfavoráveis e não foram submetidos à troca do cateter. Em 13 pacientes com mau funcionamento, a trombose da ponta do cateter foi identificada como a causa. Entre as variáveis analisadas, apenas a necessidade de intubação orotraqueal (p = 0,009), trombose venosa profunda (p = 0,01) e história de cateter anterior (p = 0,002) mostraram-se correlacionadas a um maior risco de mau funcionamento.

**Conclusões:**

A taxa de disfunção de cateter temporário de alto fluxo em pacientes com COVID-19 nesta amostra foi semelhante à taxa descrita na literatura em pacientes sem essa doença.

## INTRODUCTION

Coronavirus disease 2019 (COVID-19) was first identified in Wuhan, China, in December 2019. In March 2020, the World Health Organization (WHO) declared the outbreak a pandemic.^1^ The first confirmed case in Brazil was reported in São Paulo in February 2020. One year later, Brazil had recorded approximately 9.4 million cases, ranking third globally in total case count.^2,3^

COVID-19 can cause a wide range of symptoms, from mild to severe. While most cases are not severe,^4^ there is a high risk of thrombosis affecting both the arteries and veins, especially in severe cases that require admission to the ICU.^5^ The infection also increases the risk of acute kidney injury (AKI) and the subsequent need for hemodialysis. Furthermore, AKI increases the risk of thromboembolic events.^6^

For patients who require hemodialysis due to acute renal failure, establishing vascular access with temporary hemodialysis catheters (THC) is critical. This is essential because of the condition’s potential reversibility and the urgency of the need for treatment.^7^

Complications may arise following implantation of a THC, such as infection, dislodgement, and malfunction due to thrombosis.^8,9^ To date, three North American studies have reported an increased risk of catheter-related malfunction in hemodialysis patients with COVID-19.^10-12^

Retrospective analyses by Kanitra et al.,^12^ Shanmugasundaram et al.,^10^ and Ouyang et al.^11^ have assessed hemodialysis catheter malfunction in COVID-19 patients. Kanitra et al.^12^ evaluated malfunction of hemodialysis catheters in patients with COVID-19, reporting a malfunction rate of 31.3% in a study involving 48 patients. Shanmugasundaram et al.^10^ found a rate of 23.4% among 64 patients, while Ouyang et al.^11^ identified a dysfunction rate of 22,6%, primarily due to thrombosis, in a sample of 109 patients.

Although these studies indicate a high incidence of THC dysfunction in patients with COVID-19 and AKI, no other studies were found conducted in other populations worldwide, including in Latin America.

This study aimed to analyze the functioning of temporary hemodialysis accesses and associated complications in a Brazilian hospital treating a population infected with COVID-19.

## MATERIALS AND METHODS

This was an observational, retrospective study conducted at a secondary referral hospital in Brazil during the COVID-19 pandemic. From May to July 2020, the vascular surgery team performed and documented all THC insertions. This study presents a descriptive analysis of patients with acute kidney injury and COVID-19 during the pandemic. All COVID-19 patients over the age of 18 years who had undergone THC implantation were included in the study.

No control group was included for comparison, because the hospital where the study was conducted had been designated as a referral center exclusively for COVID-19.

The sample size was determined based on an estimated malfunction rate of 25% in international literature, with an acceptable margin of error of 10%, a significance level of 0.05, and a 95% confidence level, resulting in a minimum requirement of 73 patients. Given that the study population exceeded this calculated sample size, a sample of 107 patients was selected.

Our institution’s Ethics Committee and review board approved the study protocol, which was conducted according to the Declaration of Helsinki. It was registered under number 39458720.8.0000.0071 on the Brazilian Ethics Platform, with ethics committee consolidated opinion number 7.312.272. The study is reported according to the STROBE criteria.^13^

A vascular surgery team member carried out the implantation procedures at the bedside. Local anesthesia was administered and all punctures were guided by ultrasound. The THC used were Biomedical ® Polyurethane Double Lumen catheters, sizes 11Fr × 15cm, 11Fr × 20cm, and 12Fr × 25cm. The majority of patients (86.9%) had their catheters placed in the internal jugular vein (IJV), while 14 (13.1%) had them inserted into the common femoral vein (CFV).

After the catheter was implanted, both the flow and reflux of the catheter lines were tested to ensure they were functioning correctly. Before use, a chest X-ray was used to confirm that the catheter was positioned at the cavoatrial junction or within the right atrium or inferior vena cava.

Following the procedure, sterile dressings were applied to the puncture site. The catheters were filled with a diluted unfractionated heparin and saline solution (NaCl 0.9%) at a concentration of 50 IU/mL, following the manufacturer’s recommended volume for each pathway.

All catheters demonstrated adequate flow and reflux after insertion and were cleared for use for hemodialysis. Once implanted, the system was immediately ready for use. No complications related to device implantation were documented during or after the procedures.

During patient follow-up, the nursing team changed the dressings daily using an aseptic technique and checked for signs of inflammation at the catheter site.

Patients were followed up for a period of 7 days. The primary outcome analyzed was the incidence of THC malfunction, defined as inability to perform hemodialysis due to the impossibility of aspiration and injection through the catheter routes.

The study’s secondary outcome was to identify risk factors associated with catheter malfunction. The variables assessed included BMI, need for orotracheal intubation (OTI), hemodynamic instability requiring vasoactive drugs, anticoagulation, catheter positioning, catheter-related deep vein thrombosis (DVT), and D-Dimer levels.

To assess catheter-related DVT, the vascular surgery team performed daily Doppler ultrasound evaluations at the puncture site vein, screening for thrombosis.

Statistical analysis comparing the groups was conducted using Fisher’s exact test for categorical variables and Student’s t-test for continuous variables. A p-value of less than 0.05 was considered statistically significant for all tests. SPSS 20.0 for Windows (IBM Corp, Armonk, NY) was used to conduct the analyses.

## RESULTS

**Figure 1:**
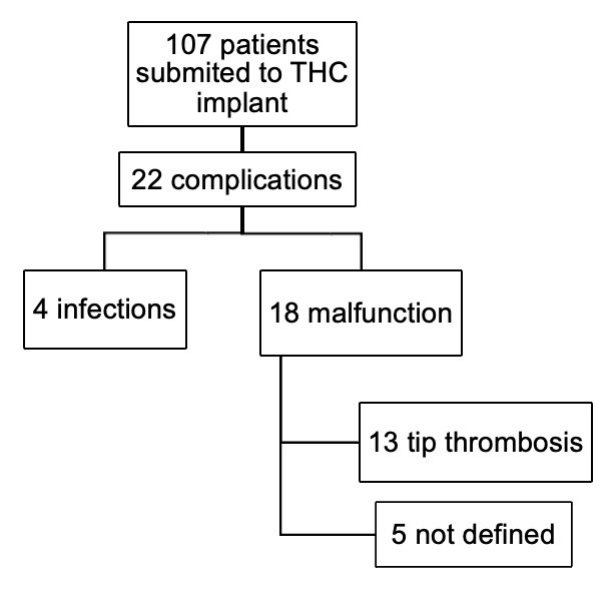
Patient enrolment flowchart. THC: Temporary hemodialysis catheter.

A total of 107 THC were implanted, as illustrated in Figure 1, which shows the patient enrollment flowchart.

Demographic and technical characteristics of the patients are detailed in Table 1. Of the 107 implanted catheters, 22 (20.6%) exhibited symptomatic complications. Specifically, 18 catheters (16.82%) malfunctioned and 4 were associated with infections. Table 2 outlines the complications related to catheter malfunction and their outcomes.

**Table 1:** Demographic and technical characteristics of patients.

Variant	Average / (*n*)	%
Age	58.2 ± 14.75 (43-84)	
Sex		
Men	69/107	64.5%
Women	38/107	35.5%
BMI	30.9 ± 7.9kg/m^2^ (19.6-69.3)	
OTI	26/107	24.3%
Hemodynamic instability	72/107	67.2%
Puncture Site		
Jugular	93/107	86.9%
Femoral	14/107	13.1%
D-Dimer	4,421 ± 2,691 ng/mL (462-8,000 ng/mL)	

BMI: body mass index; OTI: orotracheal intubation.

**Table 2:** Complications and treatments.

Variant	*n*	%
Complications	22	20.6%
Malfunction	18	16.8%
Infection	4	3.7%
Catheter replacement	17	15.8%
Clinical status prevented replacement	5	4.6%

Of the 18 malfunctioning catheters, 13 (12.14%) were attributed to obstruction caused by thrombosis. A clot was found at the tip of these catheters during device replacement, which was removed and replaced. The remaining 5 patients experienced similar obstruction issues and required catheter replacements, but were not in a suitable clinical condition to undergo the procedure and unfortunately passed away during follow-up.

The four patients who developed catheter infections presented symptoms of bacteremia during hemodialysis sessions, including worsening hemodynamic parameters, fever, and chills. The infected catheters were therefore removed and replaced.

Thirty-eight patients (35.51%) developed DVT at the site of the venous puncture. Of these, 34 patients had adequate clinical status and were fully anticoagulated for a duration of at least 3 to 5 days. The other four patients did not receive anticoagulation due to a high risk of bleeding. Thirteen patients with DVT at the puncture site had a THC malfunction.

We found no statistically significant correlation between catheter malfunction and age (p = 0.87) or BMI (p = 0.45). Additionally, there was no correlation between catheter malfunction and the access site (p = 0.292) or hemodynamic instability (p 0.10). However, significant correlations were observed between catheter malfunction and history of previous catheterization at the same puncture site (p = 0.002) and with increased clinical severity that necessitated OTI (p 0.009).

There was a significant correlation between intraluminal catheter thrombosis and malfunction (p = 0.01). However, there was no correlation between full anticoagulation and lower catheter failure rates (p = 0.628).

The mean D-Dimer value in the sample was 4,421 ± 2,691 ng/mL (462-8,000 ng/mL), and this value did not correlate with catheter dysfunction or venous thrombosis (p = 0.09). It is important to note that only 62 out of 107 patients had their D-Dimer levels assayed during the follow-up period.

## DISCUSSION

Patients with COVID-19 are at increased risk of clinical complications related to both venous and arterial thrombosis.^14^ They are also more likely to develop acute renal dysfunction requiring hemodialysis, particularly in severe cases.^15^

Implantation of a THC is generally the first option for severely ill patients who develop AKI and require hemodialysis. Device-related complications occur more frequently in this population compared to non-critical patients.^11,16-19^ In our sample, we found that patients showing signs of clinical severity, including the need for OTI, had a significantly higher risk of hemodialysis catheter malfunction (p = 0.009).

The underlying reasons for this increased risk are related to the heightened inflammatory response in these patients, which can lead to a greater risk of thromboembolic complications.^18,20^ In addition to the thrombogenic effects of COVID-19 and of AKI itself, we observed that the combination of these factors contributed to a higher incidence of catheter malfunction.

In a retrospective study involving 77 implanted THCs in patients without COVID-19, Jones and Frusha^21^ reported a malfunction rate of 23.37% and a catheter-related thrombosis rate of 11.68%. Additionally, Ouyang et al.^11^ also analyzed a population without COVID-19, and found an 8.2% rate of dialysis access malfunction, which included both catheters and arteriovenous fistulas. Furthermore, among hemodialysis patients with chronic kidney disease, also without COVID-19, those with long-term catheters exhibited a 7.1% malfunction rate.^22^

In our sample, we observed a 16.8% prevalence of malfunction. There is considerable variability in the literature regarding the frequency of THC malfunction in patients without COVID-19. However, our findings did not exceed the rates reported for similar populations without COVID-19.

In contrast, studies from North America have shown frequencies of catheter malfunction in COVID-19 patients ranging from 22.6% to 31.3%,^10-12^ This range is significantly higher than the 16.8% malfunction rate observed in our study population.

Elderly and obese patients are at greater risk of complications related to COVID-19, including thrombosis and acute renal failure.^23-25^ In our analysis, we found no relationship between age and catheter malfunction. Other studies evaluating the risk of catheter thrombosis in hemodialysis patients with COVID-19 also did not identify advanced age as a significant risk factor for catheter malfunction.^10-12^

In a retrospective analysis, Ouyang et al.^11^ analyzed THC function in COVID-19 patients and found that there was a higher rate of THC malfunction in obese patients (mean BMI: 33.1; p<0.001). Although the majority of the patients in our analysis were obese, we did not identify any factors that were associated with an increased risk of catheter malfunction or infection in this population.

The preferred access puncture site for the majority of THCs inserted at our service is the internal jugular vein, which was successfully utilized in 93 of our patients (86.9%). This vein provides a safe route directly to the right atrium. Accessing the vein requires minimal tissue manipulation and, when guided by ultrasound, it facilitates a secure and straightforward procedure, reducing the risk of complications.^26^

For catheters placed in the internal jugular vein, it is recommended to position the tip at the cavoatrial junction or even within the atrium itself. This positioning helps minimize formation of a fibrin cap.^19,27^ In the case of femoral catheters, the recommended positioning for the catheter tip is in the inferior vena cava, as this location ensures adequate flow and reflux through the pathways. This is typically achieved with catheters that are longer than 24 cm.^26,28^

Upon evaluating the relationship between device positioning and the risk of malfunction, we found no significant correlation between these two variables (p = 0.37).

Our analysis indicated that a history of central venous access was correlated with THC malfunction. As is well known, endothelial injury caused by catheter implantation, along with morphological changes in the vessel wall due to local trauma from the insertion of an intravascular device, may contribute to subsequent catheter failure.^19^ Therefore, it is crucial to preserve puncture sites before catheter implantation, particularly in patients undergoing chronic hemodialysis.

Thirteen of the 22 patients who experienced complications related to the device had malfunctions caused by thrombi at the tip of the catheter, leading to flow obstruction. Analyzing the 38 patients with DVT related to the puncture site, we found a significant correlation between thrombosis and catheter malfunction (p = 0.01). These findings align with existing literature, which indicates that patients with severe conditions and a higher risk of thrombosis tend to have higher rates of catheter thrombosis, ultimately resulting in malfunction.^29,30^

The standardized prophylactic measures established by the service included heparinizing the catheter lines using an unfractionated heparin solution. Studies have identified a lower chance of catheter thrombosis when heparinization of hemodialysis catheter lines is performed, compared to full anticoagulation and to prophylactic subcutaneous administration in the COVID-19 and hemodialysis population.^9,12^

Finally, despite elevated levels of D-dimer, this marker was not related to the prevalence of catheter dysfunction and/or venous thrombosis. This indicates that although D-dimer can be useful in predicting overall prognosis, it is not directly applicable to catheter use. This finding was corroborated by Ouyang et al.,^11^ who also found no correlation between D-Dimer levels and THC function.

### Limitations

Our study has several limitations. Since the hospital where the study was conducted only treated COVID-19 cases, we were unable to include a control group of patients without COVID-19 for comparison. Consequently, we conducted a descriptive study of THC functioning. We did not assess other comorbidities or the patients’ epidemiological data, which may have introduced selection bias, as other diseases can increase the risk of thrombosis.

Furthermore, by evaluating only the first seven days after catheter implantation, we were unable to analyze devices that could have malfunctioned after this period. This limitation could result in cases of undiagnosed malfunctions that were not considered in the study. Additionally, five of the 18 patients who experienced malfunctions died before the device could be replaced, precluding documentation of the presence or absence of thrombosis at the catheter tip.

Despite these limitations, we present the only study evaluating the function of THCs in patients with AKI and COVID-19 outside of North America, adding essential information on this important topic. Localized research is crucial because of the unique interplay of social, economic, political, and cultural factors that differ between nations and can influence study outcomes. Additionally, historical contexts and institutional structures can affect the applicability of findings across regions. Genetic diversity also contributes to population-specific health variations. By conducting country-specific studies, we can enhance local decision-making and provide valuable data for global comparisons and benchmarking. This approach improves the understanding of human diversity and the effectiveness of interventions across different contexts.

## CONCLUSION

The rate of temporary high-flow catheter dysfunction in COVID-19 patients in this sample was similar to the rate described in the literature for patients without this disease. Key predictors of catheter malfunction included prior catheter use, the need for OTI, and venous thrombosis.
